# Unraveling the Limits of Mitochondrial Control Region to Estimate the Fine Scale Population Genetic Differentiation in Anadromous Fish* Tenualosa ilisha*

**DOI:** 10.1155/2016/2035240

**Published:** 2016-05-25

**Authors:** Rashmi Verma, Mahender Singh, Sudhir Kumar

**Affiliations:** ^1^National Bureau of Fish Genetic Resources, Lucknow, Uttar Pradesh 226 002, India; ^2^University of Lucknow, Lucknow, Uttar Pradesh 226 007, India

## Abstract

The mitochondrial control region has been the first choice for examining the population structure but hypervariability and homoplasy have reduced its suitability. We analysed eight populations using control region for examining the population structure of* Hilsa*. Although the control region analysis revealed broad structuring between the Arabian Sea and Bay of Bengal (*F*_ST_  0.0441, *p* < 0.001) it was unable to detect structure among riverine populations. These results suggest that the markers used must be able to distinguish populations and control region has led to an underestimation of genetic differentiation among populations of* Hilsa*.

## 1. Introduction

The* Hilsa* shad,* Tenualosa ilisha*, is an anadromous fish with broad distribution ranging from foreshore areas, estuaries, brackish water lakes, and freshwater rivers Indo-West Pacific region from the Persian Gulf, along the coast of Pakistan, India, Bangladesh, and Burma to South Vietnam [1]. It ascends the rivers for breeding during the monsoon season and returns to the sea after completion of spawning. There has been increased exploitation on* Hilsa* fisheries in east and west coast of India over the years because of the introduction of efficient mechanized crafts such as moshari (mosquito net) seine net, behundi jal (set bag net), and char ghera jal (fence-like net operation around the char) and makeshift gears and fishers are tempted to exploit* Hilsa* stocks without caring for size and season. Overfishing may reduce population sizes to a level at which inbreeding and loss of genetic diversity occur and may result in extinction of local populations [2]. Proper scientific and judicious management actions by assessing the genetic make-up and variability of fish stock must be taken to ensure sustainability of* Hilsa* population.

The rate of evolution of mitochondrial DNA is generally higher than that of nuclear genes due to the lack of a known repair mechanism for mutations that arise during replication [3]. The control region (about 1 KB) has role in initiation of replication and transcription and is the marker of choice to identify population connectivity, conservation units, and migration routes. It has been used in many phylogenetic and population genetic studies due to high copy number and high mutation rate, as well as its maternal and haploid mode of inheritance [4]. However high evolutionary rate that has made the control region an attractive marker for biologists may be masking the true relationships between populations due to high haplotype diversity as well as homoplasy. So, an alternative marker with a slower evolutionary rate may be more suitable than the control region to reveal the population structure of* Hilsa* [5].

The population structuring of* Hilsa* was investigated by various researchers using morphomeristical, biochemical, and molecular approaches and also they differentiated three stocks of* Hilsa* belonging to Hooghly, Padma, and Ganga rivers using biometrical parameters. Based on tagging experiment, Pillay et al. [6] concluded that the same* Hilsa* individuals come up the Hooghly River during subsequent seasons, that is, winter and monsoon. Ghosh et al. [7] differentiated* Hilsa* into slender and broad morphotypes using morphometric data. Brahmane et al. [8, 9] reported more than one stock of* Hilsa* from India using RAPD markers and cytochrome b region. So, the baseline information on genetic stocks needs to be authenticated. However several morphometric and molecular studies (RAPD, RFLP) were conducted in Bangladesh and India but no study was done using mitochondrial D-loop to understand population genetic structure and patterns of gene flow of* T. ilisha* [10].

## 2. Materials and Methods

### 2.1. Fish Sampling

The present study included 77 specimens of* T. ilisha* from the geographical distribution range in India, namely, rivers draining in Bay of Bengal (i.e., Ganga, Hooghly, and Godavari) as well as from Diamond Harbour and Paradip Port and rivers draining to Arabian Sea (i.e., Narmada and Tapti).* T. ilisha* was identified and discriminated from* T. toli* and* Hilsa kelee* based on morphometric and meristic data following Talwar and Jhingran [11] and Fisher and Whitehead [12]. The* Hilsa* body is oblong and compressed with 30–33 spines like scutes on abdomen. Difference between two major* Hilsa* species, that is,* T. ilisha* and* T. toli*, is very minute. In the former, dorsal and ventral profile of the body is equally convex, while in the later abdominal profile is more convex than that of dorsal. Further, about 150 to 200 straight to slightly curved gill rakers are present on the lower part of first arch in* T. ilisha*, while in* T. toli* gill rakers are curved and the number of gill rakers is 80 to 90 as discussed by Huda and Haque [13]. The fish specimens were photographed on graph papers and meristic counts of the specimens were compared. Muscle and fin tissues were preserved in 95% v/v ethanol and the vouchers were kept in 10% v/v formaldehyde. Specific and unambiguous code was given to tissue samples and voucher of each fish specimen (Table 1).

### 2.2. DNA Extraction, PCR Amplification, and DNA Sequencing

Approximately 50 mg of caudal or anal fin or muscle tissue was used for DNA isolation following standard phenol : chloroform : isoamyl alcohol method [14]. Precipitated DNA was resuspended in TE buffer (10 mM Tris-HCl, 0.1 mM EDTA, and pH 8) and concentration was determined using Nanodrop 2000 (Thermo Scientific, USA). The primers TIDF (5′-AACTTCCACCCCTAACTCCC-3′) and TIDR (5′-GTGCTTGCGGGGCTTG-3′) were designed using Primer3 [15] and BLASTn [16] software of NCBI, so as to amplify complete control region of mitochondrial DNA (mtDNA). The PCR reaction of 50 *μ*L volume contained 1x buffer, 100 *μ*M dNTPs, 2 mM MgCl_2_, 10 picomoles of each primer, 3 U* Taq* DNA polymerase, and 100 ng template DNA. Amplifications were performed in Veriti 96 fast thermal cycler (Applied Biosystems, Inc., USA). The thermal regime for control region consisted of initial denaturation of 3 min at 94°C, followed by 35 cycles of denaturation at 94°C for 50 sec, annealing at 47°C for 30 sec, and extension at 72°C for 80 sec with final extension of 10 min at 72°C. PCR products were visualized on 1% agarose gels stained with ethidium bromide and documented using a gel documentation system (UVP, USA). DNA sequencing was performed following the dideoxynucleotide chain termination method [17] using an automated ABI 3730 sequencer (Applied Biosystems, Inc., USA).

### 2.3. Sequence Analysis

Complete control region sequence was generated from forward and reverse sequence reads using MEGA 5.1 [18]. Ambiguities were referred against the sequencing electropherograms. The consensus sequences were blasted in NCBI for the nearest similar sequence matches using BLASTn and submitted to GenBank (Table 1). DNA sequences were analysed by ClustalW, Arlequin version 3.5 [19], DnaSP version 5.10 [20], and MEGA 5.1 software for nucleotide composition, number of polymorphic sites (*S*), haplotype diversity (*h*), and nucleotide diversity (*π*).

The evolutionary history was inferred using the ML method. The bootstrap consensus tree inferred from 1000 replicates is taken to represent the evolutionary history of the taxa analysed. The evolutionary distances were computed using the Kimura 2-parameter method [21] and are in the units of the number of base substitutions per site. The rate variation among sites was modelled with a gamma distribution (shape parameter = 1).

### 2.4. Haplotype Analysis

Minimum spanning network of haplotypes was prepared by Network 4.6 software [22]. Intrapopulation diversity was analysed by estimating haplotype diversity, which indicates the probability that two randomly chosen haplotypes are different, and nucleotide diversity, which indicates the probability that two randomly chosen homologous nucleotides are different.

### 2.5. Population Genetic Analysis

The isolation-by-distance effects on population genetic structure were estimated by IBDWS 3.23 and pairwise *F*_ST_ statistics [23] using Arlequin 3.5. The hierarchical nesting of genetic diversity was estimated using the analysis of molecular variance (AMOVA) approach and was calculated using Arlequin 3.5. Significance of pairwise population comparisons was tested by 20,000 permutations. The AMOVA tests were organized in a hierarchical manner, and 1,000 permutation procedures were used to construct null distributions and to test the significance of variance components [24]. To detect population expansion or contraction, Tajima's* D* and Fu's* FS* values were estimated based on pairwise differences between sequences. Tajima's* D* test [25] calculates the distribution of allele frequency of segregating sites, whereas Fu's* FS* test [26] is based on the distribution of alleles or haplotypes.

## 3. Results

### 3.1. Molecular Characterization and Genetic Diversity

Table 1 shows the number of samples (*N*), number of haplotypes (*N*Hap), haplotype diversity (Hap*D*), and nucleotide diversity (*π*) for each population. A total of 77 individuals were sequenced for the mtDNA control region (873 bp).

Among 77 samples, 94 polymorphic sites and 58 haplotypes were detected. These polymorphisms included 64 parsimony informative sites and 32 singleton sites. The nucleotide composition (%) was 31.7 (A), 27.9 (T), 23.7 (C), and 16.7 (G).

The haplotype diversity (*h*) of the analysed populations was rather high with observed values between 1.000 ± 0.0058 in Ganga and 0.756 ± 0.01678 in Tapti population. The nucleotide diversity (*π*) within each population was very low, ranging from 0.00935 ± 0.0000012 in Hooghly to 0.01835 ± 0.0000059 in Tapti population (Table 1).

### 3.2. Haplotype Distribution and Phylogenies

**Figure 1:**
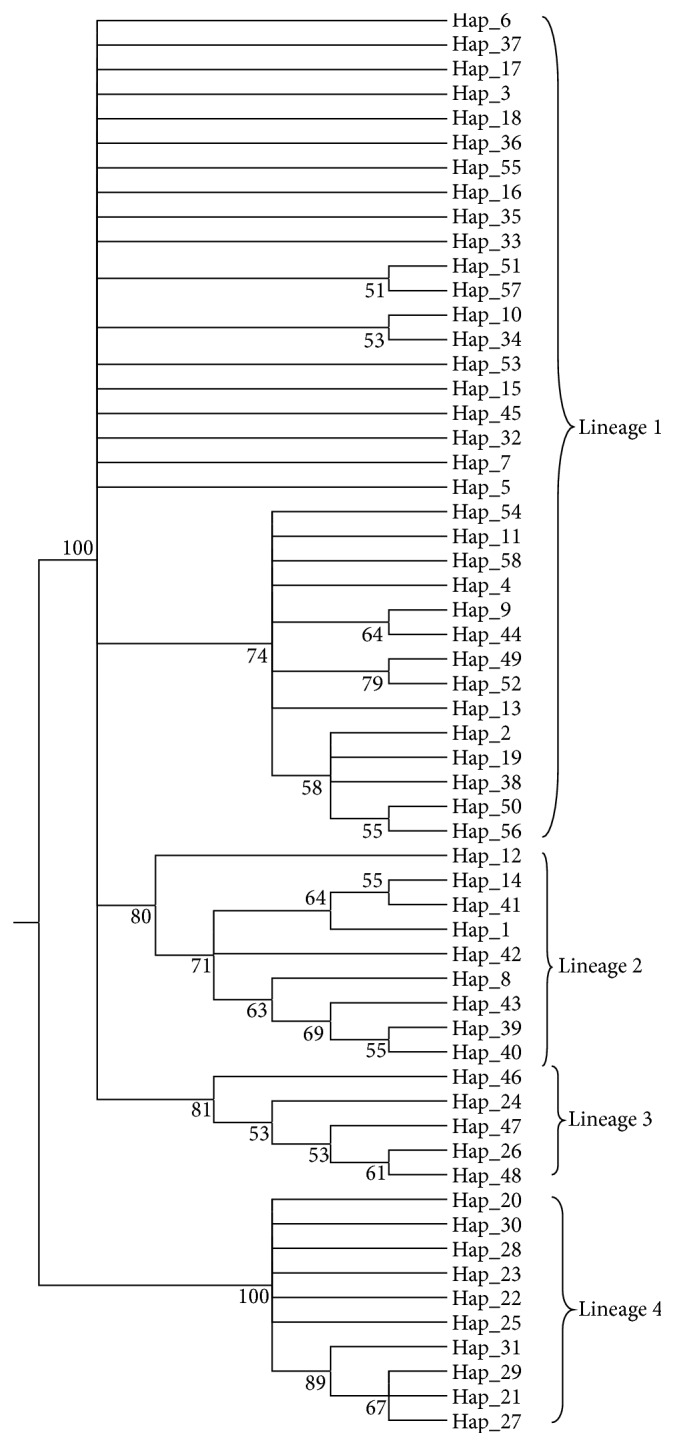
Molecular phylogenetic analysis of 58 haplotypes of* T. ilisha* constructed by Maximum Likelihood method.

**Figure 2:**
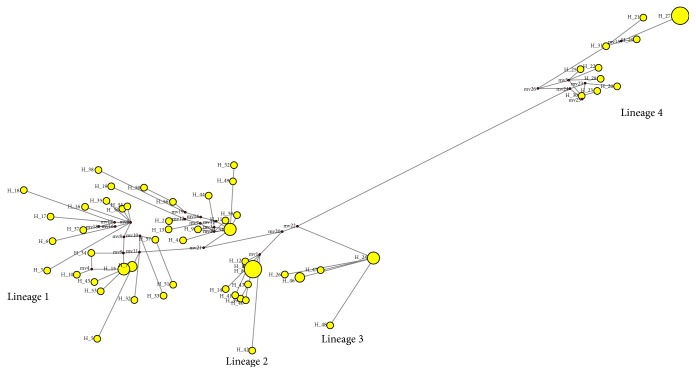
Minimum spanning network of* T. ilisha* based on D-loop haplotypes. Haplotypes separated by single lines are one mutation apart, and small circles along lines represent missing haplotypes (not sampled or extinct).

In phylogenetic study (Figure 1) and minimum spanning network of 58 haplotypes (Figure 2), eight populations of* T. ilisha* were grouped in four lineages. The haplotypes shared among different populations were 25.8%, and the rest of 74.2% were private haplotypes. Hap_8 was the most common haplotype shared among Hooghly Feeder Canal, Paradip Port, Hooghly, and Diamond Harbour populations; Hap_7 was shared among Paradip Port and Hooghly Feeder Canal populations; Hap_24 and Hap_27 were shared between Tapti and Narmada populations, Hap_1 was shared between Hooghly Feeder Canal and Ganga populations; Hap_15 was shared between Hooghly and Ganga populations (Table 2).

### 3.3. Population Genetic Analysis

There is significant geographical structuring among populations only when all populations were grouped in Bay of Bengal and Arabian Sea. AMOVA revealed 4.42% variation among populations and 95.58% variation within population (Table 3), which was further supported by significant *F*_ST_ value (i.e., 0.0441, *p* < 0.001). Estimates of genetic differentiation between eight populations using *F* statistics are given in Table 4. The populations from Narmada and Tapti showed high level of genetic differentiation from other populations.

**Figure 3:**
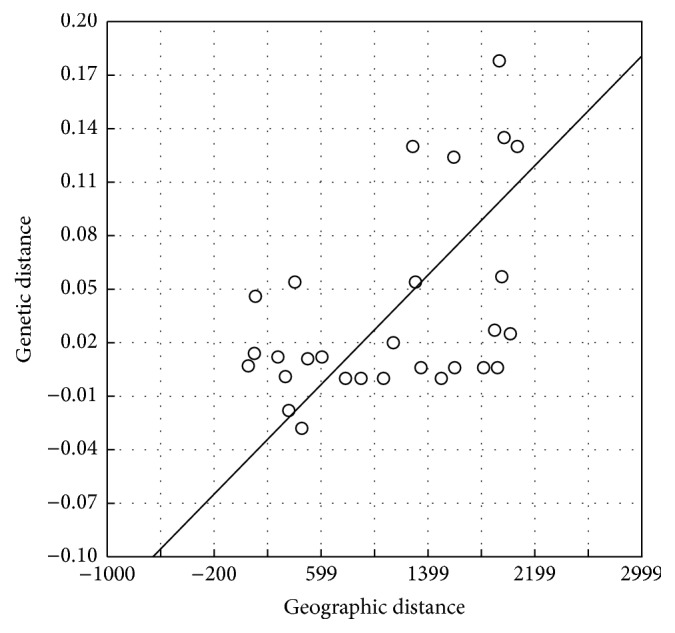
Plot of genetic distance versus geographical distance (in Kms.) for analysing isolation-by-distance patterns in* T. ilisha*.

Tajima's* D* was nonsignificantly negative for all except Narmada and Tapti populations. Fu's* FS* test also showed nonsignificant negative values for all except Diamond Harbour and Tapti populations. Large differences were observed between *θ*_0_ (population before expansion) and *θ*_1_ (population after expansion). In addition, the SSD and Hri values were also nonsignificant except for Diamond Harbour and Tapti populations (Table 5). Also, low haplotype diversity and moderate to high nucleotide diversity was observed in Diamond Harbour and Tapti populations. Mismatch distribution curves were constructed to study the genetic bottleneck and all histograms presented multimodal curves characteristic of populations with constant size over time. The plot between genetic distance and geographic distances showed a highly significant positive correlation indicating that geographic distance corroborates variation in genetic distance between* Hilsa* populations (*r* ^2^ = 0.230, *p* ≤ 0.01) (Figure 3).

## 4. Discussion

The significant positive correlation between geographical and genetic distances usually referred to as “isolation by distance” is typically suggestive of migration-drift equilibrium. Our result showed high haplotype diversity (high genetic variation) and low nucleotide diversity and significant positive Fu's* FS* and Tajima's* D* test except for Tapti and Diamond Harbour indicative of recent population expansion after a genetic bottleneck or founder events [27, 28].

In this study, *F* statistics (*F*_ST_), AMOVA, haplotype network, and phylogenetic analysis revealed genetic differentiation among the* T. ilisha* populations, suggesting that* T. ilisha* does not have a single panmictic population. Brahmane et al. [8] separated Ganga/Yamuna rivers stock from Hooghly and Narmada using RAPD markers while Brahmane et al. [9] reported low genetic diversity and absence of population differentiation of* Hilsa* by using cytochrome b region in Ganga and Hooghly rivers and also felt the need of advanced genetic markers like ATPase 8/6, nicotinamide dehydrogenase subunit 2 (ND2), and microsatellite for confirmation. Our results also strengthen the presence of more than one stock of* Hilsa* in Indian subcontinent; however Ganga and Hooghly Feeder Canal and Hooghly populations shared the same genepool. Geographical divergence and gene flow among subpopulations can be assessed by phylogenetic analysis and haplotype network provided that gene lineages have accumulated sufficient polymorphisms over time and populations have been isolated completely to allow genetic drift to act. In present study ML tree and haplotype network of control region have clearly distinguished four major clades/haplogroups, namely, Lineage 1 (Bay of Bengal populations, Diamond Harbour, Ganga, Hooghly Feeder Canal, Godavari, Paradip Port, and Hooghly), Lineage 2 (Godavari + some haplotypes from Bay of Bengal population), Lineage 3 (Tapti population), and Lineage 4 (Narmada population). Lineage 2 had mixed haplotypes mostly from Godavari and some from Hooghly Feeder Canal (Hap_1), Diamond Harbour (Hap_12), Ganga (Hap_14), and common haplotype (Hap_8); this can be explained by slightly mistaken migration routes taken by some individuals. However formation of four clades may be explained by the philopatry driven genetic differentiation that also corroborates previous tagging studies that demonstrated limited natal and breeding dispersal of* Hilsa* [6]. This type of behaviour has also been reported in highly studied American shad which exhibits a high rate of philopatry, with 97% of spawners returning to their natal stream [29]. These results were also confirmed by hierarchical AMOVA analysis, that is, among-group analysis (two-gene pool analysis of Ganga, Hooghly, Hooghly Feeder Canal, Diamond Harbour, Paradip Port, Godavari and Tapti, and Narmada gene pools) having significant variation of 3.61%. This behavioural isolation of* Hilsa* populations was also strongly supported by pairwise significant *F*_ST_ values of Tapti and Narmada with Godavari, Paradip Port, Diamond Harbour, Hooghly Feeder Canal, and Ganga. Although phylogenetic and phylogeographic analysis (minimum spanning network) shows that genetic variation is not randomly distributed among Bay of Bengal rivers, a pattern of population structure and gene flow has been difficult to verify statistically. Analysis of pairwise population *F*_ST_ provides strong support for divergence between Bay of Bengal and Arabian Sea population; however biologically accepted level of statistical significance (*p* ≤ 0.05) is too stringent to reveal the subtle genetic differences within Bay of Bengal and Arabian Sea populations when the Bonferroni correction for multiple *p* value is applied [30].

The genetic marker most commonly used to elucidate population structure in fish has been control region and was considered sensitive enough to test for structure among most populations due to rapid accumulation of mutation in the control region as well as the simplicity of mtDNA inheritance through maternal line [3]. However its suitability is questionable at interbasin level as true relationship between populations was masked by high haplotype diversity and homoplasy. *F* statistics (*F*_ST_) and AMOVA could not clearly demarcate the population structure among populations from Bay of Bengal. Mazumder and Alam [10] used RFLP of mitochondrial D-loop region to differentiate riverine, estuarine, and marine stocks and observed significant differentiation between the riverine and marine (Cox's Bazar) populations, but not between the marine and one of the estuarine populations as electrophoretic analysis of PCR-RFLP has lower resolving power than sequencing of the same PCR product. They suggested sequencing of D-loop region and faster evolving molecular markers for population structure studies, such as microsatellite loci. Similar results were recorded for yellowfin tuna where analysis of the control region between Atlantic and Indo-Pacific populations was unable to detect structure but clearly resolved by PCR RFLP of the ATPase 6/8 and COI III genes [31].

## 5. Conclusion

The populations are supposed to be separated by the philopatric behaviour. Phylogeographical structure defined by AMOVA and region specific haplotypes distinguished populations up to sea level (Bay of Bengal and Arabian sea) but population structuring at basin level was not noticed using D-loop as a marker so we suggest the use of some moderately evolving markers such as ATPase 8/6, nicotinamide dehydrogenase subunit 2 (ND2), and microsatellites for elucidating population structure of* Hilsa*.

## Figures and Tables

**Table 1:** Detail of fish samplings, haplotype diversity, nucleotide diversity and GenBank accession numbers in *T. ilisha*.

Populations	Sample size (*N*)	Number of haplotypes (*N*Hap)	Latitude/longitude	GenBank accession numbers	Haplotype diversity (Hap*D*)	Nucleotide diversity (*π*)

Hooghly Feeder Canal, Farakka, west Bengal	8	8	24.48N/87.55E	KU214744- KU214751	1.000 ± 0.00391	0.01480 ± 0.0000027

Diamond Harbour, west Bengal	8	6	24.47N/87.55E	KU214760- KU214766	0.893 ± 0.01238	0.01009 ± 0.0000030

Ganga river, below Farakka Barrage, Malda, west Bengal	7	7	22.51N/88.22E	KU214806- KU214820	1.000 ± 0.00583	0.01629 ± 0.0000049

Narmada river, Barkal, Gujarat	13	12	22.10N/88.10E	KU214752- KU214759	0.987 ± 0.00125	0.01566 ± 0.0000066

Paradip port, Odisha	9	9	20.19N/86.36E	KU214780- KU214788	1.000 ± 0.00274	0.01204 ± 0.0000062

Godavari river, Rajahmundry, Andhra Pradesh.	8	7	16.56N/81.44E	KU214789- KU214795	1.000 ± 0.00583	0.01207 ± 0.0000068

Tapti river, Ukai dam, Surat, Gujarat	8	5	21.55N/73.25E	KU214767- KU214779	0.756 ± 0.01678	0.01835 ± 0.0000059

Hooghly, Kalyani, west Bengal	16	12	21.15N/73.35E	KU214796- KU214805	0.962 ± 0.00159	0.00935 ± 0.0000012

**Table 2:** Relative haplotype frequencies in different populations of *T. ilisha*.

Haplotype	Hooghly Feeder Canal	Diamond Harbour	Ganga	Narmada	Paradip Port	Godavari	Tapti	Hooghly
Hap_1	0.125	0	0.143	0	0	0	0	0
Hap_2	0.125	0	0	0	0	0	0	0
Hap_3	0.125	0	0	0	0	0	0	0
Hap_4	0.125	0	0	0	0	0	0	0
Hap_5	0.125	0	0	0	0	0	0	0
Hap_6	0.125	0	0	0	0	0	0	0
Hap_7	0.125	0	0	0	0.111	0	0	0
Hap_8	0.125	0.375	0	0	0.111	0	0	0.066
Hap_9	0	0.125	0	0	0	0	0	0
Hap_10	0	0.125	0	0	0	0	0	0
Hap_11	0	0.125	0	0	0	0	0	0
Hap_12	0	0.125	0	0	0	0	0	0
Hap_13	0	0.125	0	0	0	0	0	0
Hap_14	0	0	0.143	0	0	0	0	0
Hap_15	0	0	0.143	0	0	0	0	0.133
Hap_16	0	0	0.143	0	0	0	0	0
Hap_17	0	0	0.143	0	0	0	0	0
Hap_18	0	0	0.143	0	0	0	0	0
Hap_19	0	0	0.143	0	0	0	0	0
Hap_20	0	0	0	0.0769	0	0	0	0
Hap_21	0	0	0	0.0769	0	0	0	0
Hap_22	0	0	0	0.0769	0	0	0	0
Hap_23	0	0	0	0.0769	0	0	0	0
Hap_24	0	0	0	0.154	0	0	0.1	0
Hap_25	0	0	0	0.0769	0	0	0	0
Hap_26	0	0	0	0.0769	0	0	0	0
Hap_27	0	0	0	0.0769	0	0	0.5	0
Hap_28	0	0	0	0.0769	0	0	0	0
Hap_29	0	0	0	0.0769	0	0	0	0
Hap_30	0	0	0	0.0769	0	0	0	0
Hap_31	0	0	0	0.0769	0	0	0	0
Hap_32	0	0	0	0	0.111	0	0	0
Hap_33	0	0	0	0	0.111	0	0	0
Hap_34	0	0	0	0	0.111	0	0	0
Hap_35	0	0	0	0	0.111	0	0	0
Hap_36	0	0	0	0	0.111	0	0	0
Hap_37	0	0	0	0	0.111	0	0	0
Hap_38	0	0	0	0	0.111	0	0	0
Hap_39	0	0	0	0	0	0.143	0	0
Hap_40	0	0	0	0	0	0.143	0	0
Hap_41	0	0	0	0	0	0.143	0	0
Hap_42	0	0	0	0	0	0.143	0	0
Hap_43	0	0	0	0	0	0.143	0	0
Hap_44	0	0	0	0	0	0.143	0	0
Hap_45	0	0	0	0	0	0.143	0	0
Hap_46	0	0	0	0	0	0	0.2	0
Hap_47	0	0	0	0	0	0	0.1	0
Hap_48	0	0	0	0	0	0	0.1	0
Hap_49	0	0	0	0	0	0	0	0.066
Hap_50	0	0	0	0	0	0	0	0.066
Hap_51	0	0	0	0	0	0	0	0.066
Hap_52	0	0	0	0	0	0	0	0.066
Hap_53	0	0	0	0	0	0	0	0.066
Hap_54	0	0	0	0	0	0	0	0.2
Hap_55	0	0	0	0	0	0	0	0.066
Hap_56	0	0	0	0	0	0	0	0.066
Hap_57	0	0	0	0	0	0	0	0.066
Hap_58	0	0	0	0	0	0	0	0.066

**Table 3:** Hierarchal analysis of molecular variance (AMOVA) for *T. ilisha*.

Source of variation	d.f.	Sum of squares	Variance components	Percentage of variation
Among populations	7	4.773	0.02189*V*_*a*_	4.42
Within populations	69	32.681	0.47364*V*_*b*_	95.58

Total	76	37.455	0.49553	

Fixation index	*F*_ST_: 0.0441			

**Table 4:** Pairwise *F*_ST_ (below diagonal) and associated *p* values (above diagonal) among *T. ilisha *populations. Bold values denote statistical significant values after the Bonferroni correction.

	Hooghly Feeder Canal	Diamond Harbour	Ganga	Narmada	Paradip Port	Godavari	Tapti	Hooghly
Hooghly Feeder Canal	0	0.5855	0.9909	0.48649	0.99099	0.99099	0.03604	0.22523
Diamond Harbour	0.0070	0	0.16216	0.03604	0.28829	0.19820	0.00000	0.03604
Ganga	−0.0181	0.0547	0	0.50450	0.99099	0.99099	0.03604	0.53153
Narmada	0.0067	0.0570^*∗*^	0.00687	0	0.44144	0.62162	0.03604	0.03604
Paradip Port	−0.0285	0.0115	0	0.0066	0	0.99099	0.00000	0.31532
Godavari	0	0.0547	0	0.0068	0	0	0.01802	0.18018
Tapti	0.127^*∗*^	0.1788^*∗∗*^	0.1302^*∗*^	0.0749^*∗*^	0.1243^*∗∗*^	0.1302^*∗*^	0	0.00000
Hooghly	0.0120	0.0462^*∗*^	0.0015	0.0256^*∗*^	0.0126	0.0207	0.1351^*∗∗*^	0

^*∗∗*^ *p* < 0.001; ^*∗*^ *p* < 0.05.

**Table 5:** Genetic diversity indices and demographic parameters of *T. ilisha*.

Population	ti	tv	*θ*_0_	*θ*_1_	Tajima's *D*	Fu's *FS*	Hri	SSD
Hooghly	26	1	7.490	99999	−0.07622	−2.7044	0.04227	0.01401
Diamond Harbour	22	1	7.200	99999	−0.05012	0.78291	0.16709	0.10294^*∗*^
Hooghly Feeder Canal	31	6	4.826	99999	−0.51513	−1.6082	0.12245	0.04966
Paradip Port	31	6	20.202	99999	−1.15597	−2.6925	0.02623	0.02041
Godavari	22	9	2.638	99999	−0.80192	−1.4708	0.09524	0.05183
Ganga	30	6	12.179	99999	−0.19653	−0.9430	0.24943	0.07201
Narmada	37	2	17.159	201.765	0.41084	−2.3072	0.02548	0.02592
Tapti	27	6	12.237	99999	1.99567	5.55599	0.13975	0.11588^*∗*^

^*∗*^ *p* < 0.05.

## References

[1] Pillay S. R., Rosa H. (1963). Synopsis of biological data on hilsa, *Hilsa ilisha* (Hamilton 1882). *FAO Fisheries Biology Synopsis*.

[2] Lakra W. S., Mohindra V., Lal K. K. (2007). Fish genetics and conservation research in India: status and perspectives. *Fish Physiology and Biochemistry*.

[3] Avise J. C. (1994). *Molecular Markers: Natural History and Evolution*.

[4] Avise J., Arnold J., Ball R. (1987). Intraspecific phylogeography: the mitochondrial DNA bridge between population genetics and systematics. *Annual Review of Ecology, Evolution, and Systematics*.

[5] Bradman H. M., Grewe P. M., Appleton B. (2011). Direct comparison of mitochondrial markers for the analysis of swordfish population structure. *Fisheries Research*.

[6] Pillay S. R., Rao K. V., Mathur P. K. (1962). Preliminary report on the tagging of the Hilsa, *Hilsa ilisha* (Hamilton). *Proceedings of the Indo-Pacific Fisheries Council, India*.

[7] Ghosh A. N., Bhattacharya R. K., Rao K. V. (1968). On the identification of the sub-populations of *Hilsa ilisha* (Ham.) in the Gangetic system with a note on their distribution. *Proceedings of the National Academy of Sciences, India, Section B: Biological Sciences*.

[8] Brahmane M. P., Das M. K., Sinha M. R. (2006). Use of RAPD fingerprinting for delineating populations of hilsa shad *Tenualosa ilisha* (Hamilton, 1822). *Genetics and Molecular Research*.

[9] Brahmane M. P., Kundu S. N., Das M. K., Sharma A. P. (2013). Low genetic diversity and absence of population differentiation of hilsa (*Tenualosa ilisha*) revealed by mitochondrial DNA cytochrome b region in Ganga and Hooghly rivers. *African Journal of Biotechnology*.

[10] Mazumder S. K., Alam M. S. (2009). High levels of genetic variability and differentiation in hilsa shad, *Tenualosa ilisha* (Clupeidae, Clupeiformes) populations revealed by PCR-RFLP analysis of the mitochondrial DNA D-loop region. *Genetics and Molecular Biology*.

[11] Talwar K., Jhingran A. G. (1991). *Inland Fishes of India and Adjacent Countries*.

[12] Fisher W., Whitehead P. J. P. (1974). *FAO Species Identification Sheets, Fishing Area 57 and 71*.

[13] Huda S. M., Haque M. E. (2003). *Field Guide to Finfishes of Sundarban*.

[14] Sambrook J., Russell D. W. (2001). *Molecular Cloning-A Laboratory Manual*.

[15] Rozen S., Skaletsky H. J., Krawetz S., Misener S. (2000). Primer3 on the WWW for general users and for biologist programmers. *Bioinformatics Methods and Protocols: Methods in Molecular Biology*.

[16] Altschul S. F., Gish W., Miller W., Myers E. W., Lipman D. J. (1990). Basic local alignment search tool. *Journal of Molecular Biology*.

[17] Sanger F., Nicklen S., Coulson A. R. (1977). DNA sequencing with chain-terminating inhibitors. *Proceedings of the National Academy of Sciences of the United States of America*.

[18] Tamura K., Peterson D., Peterson N., Stecher G., Nei M., Kumar S. (2011). MEGA5: molecular evolutionary genetics analysis using maximum likelihood, evolutionary distance, and maximum parsimony methods. *Molecular Biology and Evolution*.

[19] Excoffier L., Lischer H. E. L. (2010). Arlequin suite ver 3.5: a new series of programs to perform population genetics analyses under Linux and Windows. *Molecular Ecology Resources*.

[20] Librado P., Rozas J. (2009). DnaSP v5: a software for comprehensive analysis of DNA polymorphism data. *Bioinformatics*.

[21] Kimura M. (1980). A simple method for estimating evolutionary rates of base substitutions through comparative studies of nucleotide sequences. *Journal of Molecular Evolution*.

[22] Bandelt H.-J., Forster P., Röhl A. (1999). Median-joining networks for inferring intraspecific phylogenies. *Molecular Biology and Evolution*.

[23] Wright S. (1965). The interpretation of population structure by F-statistics with special regard to systems of mating. *Evolution*.

[24] Guo S. W., Thompson E. A. (1992). Performing the exact test of Hardy-Weinberg proportion for multiple alleles. *Biometrics*.

[25] Tajima F. (1989). Statistical method for testing the neutral mutation hypothesis by DNA polymorphism. *Genetics*.

[26] Fu Y.-X. (1997). Statistical tests of neutrality of mutations against population growth, hitchhiking and background selection. *Genetics*.

[27] Grant W. S., Bowen B. W. (1998). Shallow population histories in deep evolutionary lineages of marine fishes: insights from sardines and anchovies and lessons for conservation. *Journal of Heredity*.

[28] Aboim M. A., Menezes G. M., Schlitt T., Rogers A. D. (2005). Genetic structure and history of populations of the deep-sea fish Helicolenus dactylopterus (Delaroche, 1809) inferred from mtDNA sequence analysis. *Molecular Ecology*.

[29] Melvin G. D., Dadswell M. J., Martin J. D. (1986). Fidelity of American shad, *Alosa sapidissima* (Clupeidae), to its river of previous spawning. *Canadian Journal of Fisheries and Aquatic Sciences*.

[30] Reeb C. A., Arcangeli L., Block B. A. (2000). Structure and migration corridors in Pacific populations of the Swordfish *Xiphius gladius*, as inferred through analyses of mitochondrial DNA. *Marine Biology*.

[31] Ely B., Viñas J., Alvarado Bremer J. R. (2005). Consequences of the historical demography on the global population structure of two highly migratory cosmopolitan marine fishes: The yellowfin tuna (Thunnus albacares) and the skipjack tuna (Katsuwonus pelamis). *BMC Evolutionary Biology*.

